# Effect of chronic low-dose treatment with chitooligosaccharides on microbial dysbiosis and inflammation associated chronic ulcerative colitis in Balb/c mice

**DOI:** 10.1007/s00210-023-02710-3

**Published:** 2023-09-11

**Authors:** K M Rajesh, Manas Kinra, Niraja Ranadive, Goutam Mohan Pawaskar, Jayesh Mudgal, Ritu Raval

**Affiliations:** 1 Department of Biotechnology, Manipal Institute of Technology, Manipal Academy of Higher Education, Manipal, Karnataka 576104 India; 2 Department of Pharmacology, Manipal College of Pharmaceutical Sciences, Manipal Academy of Higher Education, Manipal, Karnataka 576104 India

**Keywords:** Inflammatory bowel disease (IBD), Chitooligosaccharides, Gut microbiota, Inflammation, DSS colitis, BALB/c mice

## Abstract

The study aimed to investigate the potential of low dose chitooligosaccharide (COS) in ameliorating dextran sodium sulfate (DSS) induced chronic colitis by regulating microbial dysbiosis and pro-inflammatory responses. Chronic colitis was induced in BALB/c mice by DSS (4% w/v, 3 cycles of 5 days) administration. The mice were divided into four groups: vehicle, DSS, DSS + mesalamine and DSS+COS. COS and mesalamine were administered orally, daily once, from day 1 to day 30 at a dose of 20 mg/kg and 50 mg/kg respectively. The disease activity index (DAI), colon length, histopathological score, microbial composition, and pro-inflammatory cytokine expression were evaluated. COS (20 mg/kg, COS^Low^) administration reduced the disease activity index, and colon shortening, caused by DSS significantly. Furthermore, COS^Low^ restored the altered microbiome in the gut and inhibited the elevated pro-inflammatory cytokines (IL-1 and IL-6) in the colon against DSS-induced chronic colitis in mice. Moreover, COS^Low^ treatment improved the probiotic microflora thereby restoring the gut homeostasis. In conclusion, this is the first study where microbial dysbiosis and pro-inflammatory responses were modulated by chronic COS^Low^ treatment against DSS-induced chronic colitis in Balb/c mice. Therefore, COS supplementation at a relatively low dose could be efficacious for chronic inflammatory bowel disease.

**Supplementary Information:**

The online version contains supplementary material available at 10.1007/s00210-023-02710-3.

## Introduction

Chronic inflammatory gastrointestinal tract disorders fall under the umbrella term of Inflammatory Bowel Disease (IBD), which encompasses conditions such as ulcerative colitis (UC) and Crohn disease (CD) (Becker et al. 2015b). The prevalence of inflammatory bowel disease (IBD) is reported to be 396 cases per 100,000 people. Within the IBD group, ulcerative colitis (UC) is diagnosed in a range of 0.5 to 24.5 cases per 100,000 individuals annually, while Crohn's disease (CD) is diagnosed in a range of 0.1 to 16 cases per 100,000 individuals over a decade (Alatab et al. 2020). The conditions of UC and CD can be distinguished based on the location, type and inflammatory characteristics (Hendrickson et al. 2002). The occurrence of IBD in genetically susceptible person is mitigated by complex environmental, microbial, and immune-mediated interactions (Becker et al. 2015a). The intestinal microbiota and mucosal immune system are distinct in healthy subjects. This disengagement gets altered in IBD patients, thereby facilitating the interaction between intestinal microbiota the mucosal immune component (Zhang et al. 2017; Glassner et al. 2020).

The naturally occurring polymers, have displayed immunomodulatory effects. These polymers include ligstroside, hydroxytrosol and oleuropein derived from olive trees, polyphenols derived from green tea, chondroitin sulphate derived from shark cartilage, hyaluronic acid derived from eyeball and liver of swordfish, chitosan derived from arthropods (crustaceans) and alginate derived from brown seaweeds (Khare et al. 2020; Bilal et al. 2021).

Previous studies have reported the protective effects of chitosan derivative, chitooligosaccharide (COS) against ulcerative colitis in mice. Pre-treatment with COS (MW 5000 -10000 Da) for 11 days, displayed promising effects against different models of acute colitis (Yousef et al. 2012), where a 20 mg/kg dose of COS (COS^Low^) was found to be the most effective against acute colitis in mice. Compared to a high dose (100 mg/kg), COS^Low^ was reported to be more efficacious against acute colitis condition in mice. In the same study, seven- and five-day oral treatment with COS^Low^ produced better protection against DSS-induced colitis in mice (Yousef et al. 2012). In addition, five days of COS (2% w/w) feeding with diet corrected the systemic inflammation and acute colitis in mice (Azuma et al. 2015). However, studies on the administration of COS^Low^ for a time span greater than a week with respect to chronic colitis and gut dysbiosis are missing.

Recently, the mechanism of COS (1500Da) was reported via inhibition of TLR4/STAT3/NF-κB signaling pathway of chronic ulcerative colitis. However, the protective effect of COS was reported at relatively high doses (250 and 500 mg/kg) in mice (Guo et al. 2021). Thirty-seven days pretreatment with COS, protected the mice from chronic ulcerative colitis by modulation of gut microbiota dysbiosis (Guo et al. 2021). However, there is a gap in research on impact of COS^Low^ on gut microbiota dysbiosis which may provide an insight for the better efficacy of COS considering risk to benefit ratio. In another recent study, different molecular weight chitin derivatives including COS (1500 Da and 2500 Da) were tested for a span of 14 days in low DSS concentration (2.5%) induced acute ulcerative colitis (UC) in mouse model. The efficacy was compared at relatively higher doses of 50 and 200 mg/kg/day, which were found identical in improving the colitis symptoms, and restoring gut microbiota balance (Mei et al. 2022). Although, these evidence supports the protective / therapeutic effect of COS in acute and chronic colitis in mice models, but the effect of chronic (>30 days) COS^Low^ treatment is missing. To address this, the objective of the present study was to evaluate the potential of low-dose, long-term administration of COS against chronic UC. In addition, the present study investigated the impact of COS^Low^ on gut dysbiosis and gut inflammation.

## Material and methods

### Material

Chitooligosaccharides (COS, 221.21-1548.47 Da) was obtained from Tokyo Chemical Industry in Japan. Enzyme-Linked Immunosorbent Assay (ELISA) kits from ThermoFisher Scientific, USA, were purchased for interleukin-1 (IL-1) and interleukin-6 (IL-6) estimation in mice colon tissue. Dextran sodium sulphate (DSS, 36-50kDa) was obtained from Sisco Research Laboratories, India. All other chemicals were of the highest quality for laboratories.

### Animals

Thirty-two male BALB/c mice aged 6-8 weeks inbred in the Central Animal Research Facility at Manipal Academy of Higher Education (CARF, MAHE, Manipal) were used in this study. The animals were placed within plastic enclosures maintained at approximately 25°C with a variance of 0.5°C. Each enclosure held four animals with unrestricted access to food and water. The lighting followed a schedule of 12 hours of light and 12 hours of darkness, while the humidity level was kept around 50% with a potential fluctuation of 5%. All animals were cared for and handled in accordance with the Committee for Control and Supervision of Experiments on Animals guidelines. The experimental protocol was approved by Institutional Animals Ethics Committee (Approval No. IAEC/KMC/03/2021, dated 23 January 2021) Manipal Academy of Higher Education (MAHE).

### Experimental design and induction of chronic colitis in mice

**Fig. 1:**
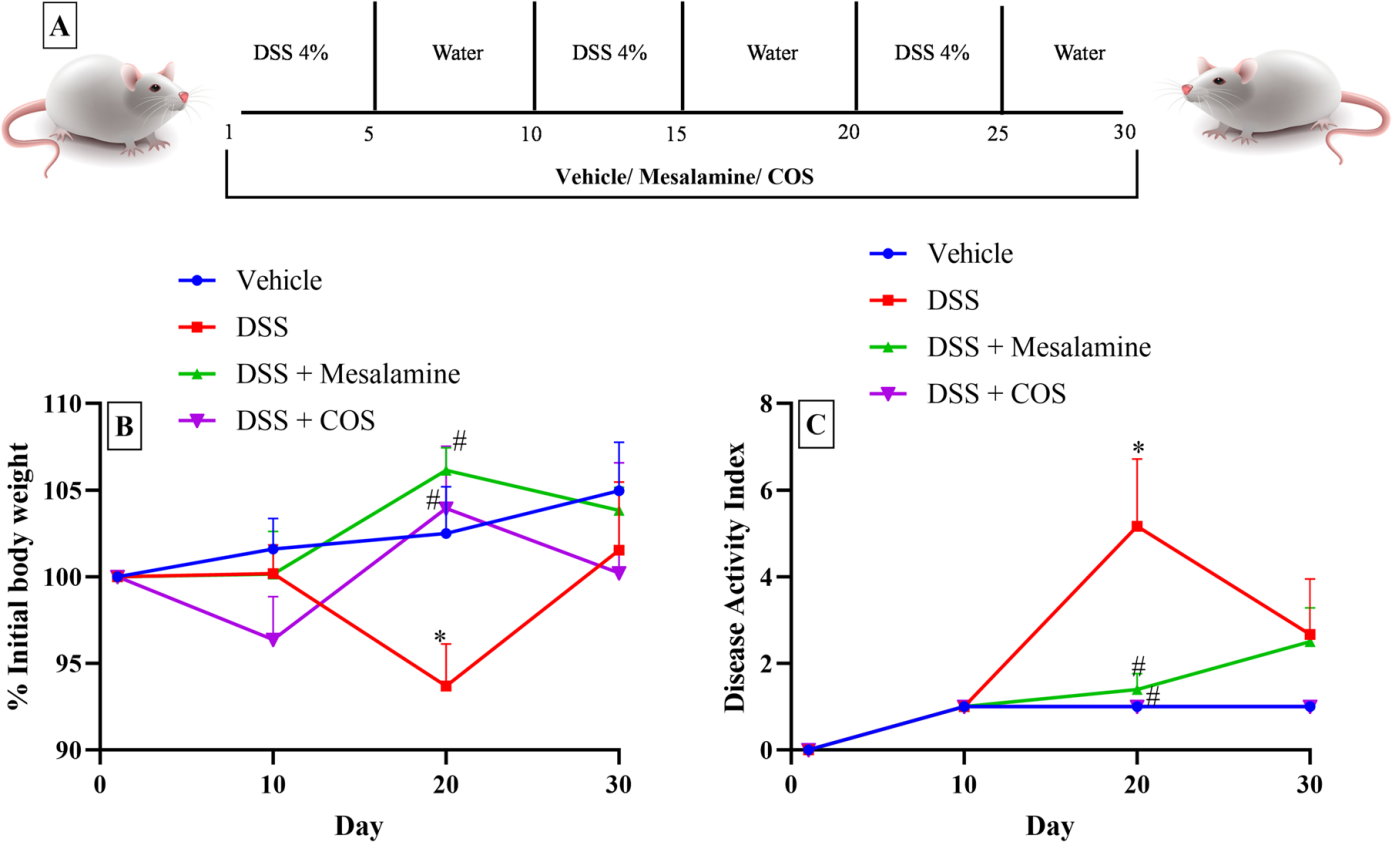
Effect of treatments on body weight and disease activity index (DAI) score against DSS-induced colitis in mice. **A**) Design of experiment (n=8), **B**) % of initial body weight (n =4-8), **C**) Disease Activity Index (DAI) (n =4-8). All the data presented as Mean ± SEM. *p<0.05 vs. vehicle and #p<0.05 vs. DSS group

The chronic colitis was induced by including DSS (4% w/v) in drinking water for 5 days, and weaning for the next 5 days. This cycle was repeated three times as depicted in figure (Fig. 1A) (Peng et al. 2020) . The mice were randomly divided into four groups (n=8). Group 1 received vehicle (water); Group 2 received DSS (4% w/v) (water + DSS); Group 3 received DSS + mesalamine (50 mg/kg, po) (water + DSS + mesalamine) and Group 4 received DSS + chitooligosaccharide (COS) (20 mg/kg, po) (water + DSS + COS). The dose of mesalamine and COS was selected based on the published literature (Mei et al. 2022). The animals were administered vehicle/mesalamine/COS orally daily once in the 30 day duration of the study. The animals were monitored daily for body weight, rectal bleeding and stool consistency (Kim et al. 2002). After the completion of the study cycle on the 30^th^ day, stool samples were collected from the colon region. The animals were euthanized, and their colons were removed and preserved at -80 ºC for subsequent analysis.

### Disease activity index

The disease activity index (DAI), which measures the severity of the disease, is frequently used in IBD animal models. Weight loss, the consistency of the stool, and the existence of stools with blood were selected as crucial colitis symptoms (Hidalgo-Cantabrana et al. 2016). Based on the percentage body weight loss, consistency of the stool, and the incidence of blood in the stool, a DAI was calculated for each animal. The percentage body weight loss score of 0 was given for no change, 1 for losses of 10% or less, 2 for 15% or more, 3 for 16–20%, and 4 for losses of more than 20%. Similarly, with respect to the stool consistency, 0 was awarded for normal, 2 for loose stool, and 4 for a diarrhoea condition. A score of 0 was assigned with no blood in stools and 4 for the presence of blood in stool (Peng et al. 2020).

### Histopathologic evaluation

At the time of sacrifice, the length and weight of each mouse were recorded, and the colon harvested. The colon tissue was stained with haematoxylin and eosin(H&E) after being fixed in 10% normal buffered formalin. All H&E-stained tissue sections were graded based on inflammatory severity (0-3), inflammation extent (0-3), and cryptitis/crypt abscess (0-3) (Dieleman et al. 1998).

### Enzyme-Linked Immunosorbent assay for gut cytokines

The colon tissue samples were homogenised in 10% v/v ice-cold phosphate buffer (100 mM, pH 7) and centrifuged at 10000 rpm for 10 minutes. The recovered supernatants were subjected for IL-1β and IL-6 determination using the ELISA kits according to the manufacturer’s protocol (Atreya and Neurath 2005; Mudter and Neurath 2007; Coccia et al. 2012; Mao et al. 2018).

### Estimation of malondialdehyde for lipid peroxidation

The lipid peroxidation was estimated in the colon tissue samples by homogenisation in 10% v/v phosphate buffer at room temperature (0.1 M and pH 7). The stock solution of TBA-TCA-HCl reagent was made by mixing 15% w/v Trichloroacetic acid (TCA), 0.375% w/v Thiobarbituric acid (TBA), and 0.25N hydrochloric acid (HCl). Equal amounts of tissue homogenate and TBA-TCA-HCl reagent were combined and heated at 90 °C for 10-15 minutes. The samples were subsequently subjected to centrifugation at 6000 rpm at 4 °C for 10 minutes. The absorbance of the supernatant was measured at 530 nm (Janero 1990; Mudgal et al. 2020).

### 16S rRNA gut microbiota analysis

16S rRNA V3-V4 hyper variable region sequencing (Clevergene, Bangalore, India) was performed on the faecal samples collected from the mice prior to the sacrifice. The sequencing employed KAPA HiFi HotStart Ready Mix kit and 341F and 785R as the primers. Sequencing libraries were created by running, an additional 8 cycles of DNA polymerase chain reaction (PCR) using Illumina bar-coded adapters. The sequencing data was generated by Illumina MiSeq software (USA). GREENGENES v.13.8-99 database, was employed to filter contigs and their organization into operational taxonomic units (OTU). The abundance of these OTU was estimated Fisher's exact test was used to determine the statistical significant differences in OTU abundance between samples (Schierová et al. 2020; Peng et al. 2020).

### Statistical analysis

All data except for body weight and DAI, were analysed using one-way ANOVA followed by post-hoc Tukey's test in GraphPad Prism version 8.0.0. The statistical relevance of body weight and DAI was performed by Tukey's multiple comparison. The data is represented as mean ± SEM. The symbols (*) and (#) signify p<0.05 when compared to the vehicle and DSS groups, respectively.

## Results

### Chronic COS^Low^ prevented body weight loss and disease activity against DSS-induced chronic colitis in mice

The experimental regime followed for the study is depicted in Fig. 1A. The figure compares DSS (4%)-induced colitis, altered body weight and DAI with respect to the baseline. As compared to the vehicle-treated group, the body weight and DAI of the DSS group had significantly (p<0.05) declined on day 20 (Fig. 1). The treatment with mesalamine (50 mg/kg) and Cos^Low^ reversed the changes in the body weight and DAI observed after DSS administration (Fig. 1B and C). On day 30, there were no significant body weight / DAI difference between the groups.

### Chronic COS^Low^ treatment prevented DSS-induced gross changes in colon

**Fig. 2:**
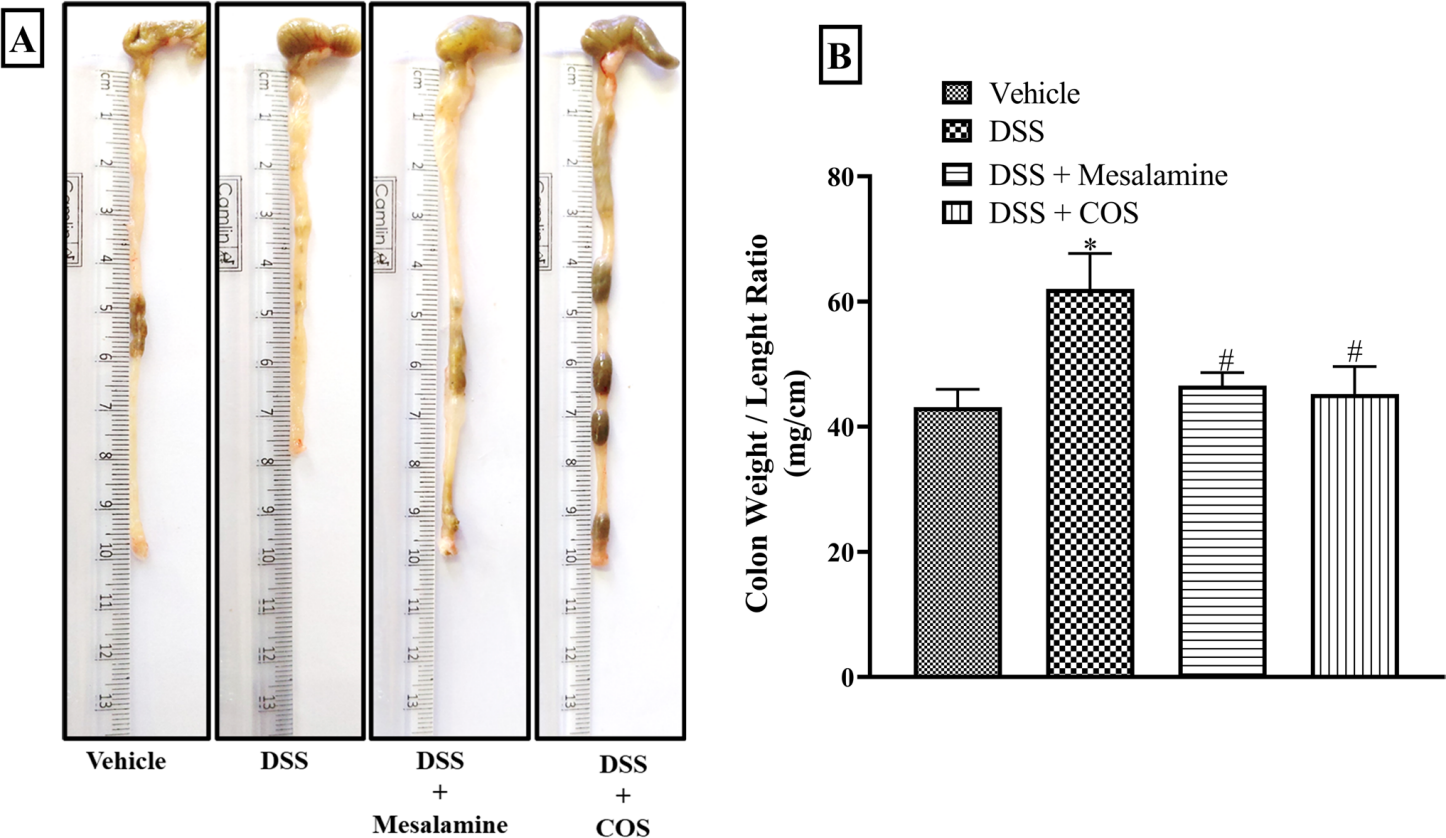
Effect of COS on DSS-induced gross changes in colon. **A**) Representative images of colon and **B**) colon weight/length ratio. All data are presented as mean ± SEM (n=4-8), *p<0.05 vs. vehicle and #p<0.05 vs. DSS group

Increased colon weight/length ratio represents granulomatous inflammation, a characteristic sign of chronic colitis (Sydora et al. 2012; Chassaing et al. 2014a). Administration of DSS for a period of thirty days led to a significant (p<0.05) increase in colon weight/length ratio as compared to the vehicle group (Fig. 2A and B). Pre-treatment with mesalamine and COS^Low^ significantly reduced the ratio, indicating protection imparted by these compounds against DSS-mediated chronic colitis (Fig. 2A and B).

### Effect of chronic COS^Low^ treatment on DSS-induced histopathological changes in colon

**Fig. 3:**
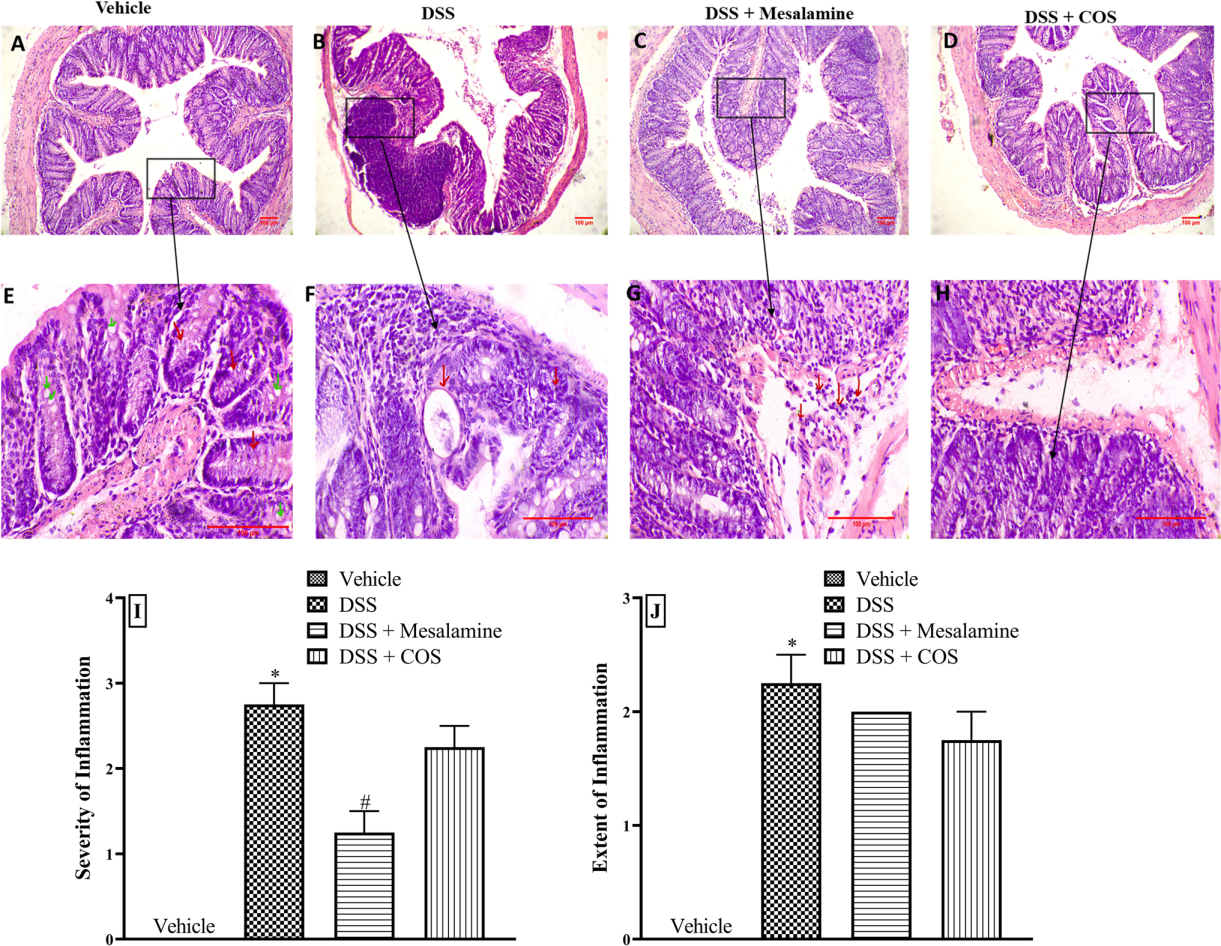
Effect of COS on DSS-induced histological changes in the colon (n = 2). Tissue section (100×) showing **A**) thick mucosa, submucosa and muscularis layers in the vehicle, **B**) chronic and acute inflammatory infiltrates in DSS, **C**) reduction of inflammatory cells in DSS + mesalamine and **D**) reduction of acute inflammatory cells in DSS + COS, groups respectively. Tissue section (400×) showing **E**) mucosa with crypts (red) and goblet cells (green) in the vehicle, **F**) chronic and acute inflammatory infiltrate with dilated crypt in DSS, **G**) chronic inflammatory cells in submucosa in DSS + mesalamine and **H**) reduced acute inflammatory cells in DSS + COS, groups respectively. **I**) Severity of inflammation. **J**) Extent of inflammation. All data are presented as mean ± SEM, *p<0.05 vs. vehicle and #p<0.05 vs. DSS group

As compared to the vehicle group (Fig. 3A and E), the colon histology of DSS-administered mice showed the presence of inflammatory cells such as neutrophils in the mucosa, submucosa and muscularis layers. Cryptitis and reduced goblets cells were also confirmed in the colon of DSS-administered mice confirming the chronic colitis (Fig. 3B and F). These histological changes, involving inflammatory infiltration, were reduced in the mesalamine and COS^Low^ group (Fig. 3C-D and G-H). In the DSS group, scores for the intensity and extent of inflammation were significantly (p<0.05) higher than in the vehicle-treated group. Although there was a gross reduction in the severity and amount of inflammation in the mesalamine and COS^Low^ treated group, this impact was statistically not significant (Fig. 3I-J).

### Chronic COS^Low^ treatment is able to prevent DSS-induced colon cytokines

**Fig. 4:**
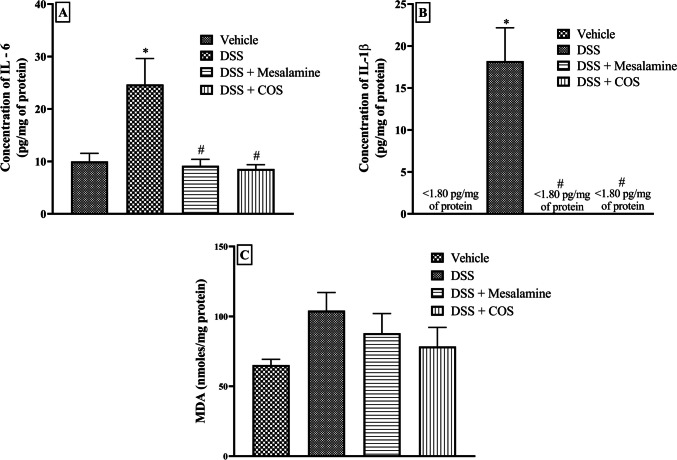
Effect of COS on DSS-induced colon inflammatory markers. **A**) IL-6 (n = 3-4), **B**) IL-1β (n = 3-6) and **C**) Malondialdehyde (MDA) levels (n = 3-4). All data are presented as mean ± SEM, *p<0.05 vs. vehicle and #p<0.05 vs. DSS group

An upsurge in the pro-inflammatory cytokines such as IL-6 and IL-1β in the colitis condition has been linked with chronic colitis (McLean et al. 2014). The administration of DSS led to a significant (p<0.05) increase in the colon IL-6 and IL-1β levels in DSS group animals as compared with vehicle group (Fig. 4A and B). However, pre-treatment with mesalamine and COS^Low^ significantly reduced (p<0.05) the elevated levels of these proinflammatory cytokines in the colon, indicating anti-inflammatory protection against DSS-mediated chronic colitis. However, there was no significant increase in malondialdehyde (MDA), an oxidative stress marker, in DSS group animals, and the effects mesalamine and COS^Low^ could not be established (Fig. 4C).

### Chronic COS^Low^ treatment increased the diversity of gut microbiota against DSS-induced chronic colitis in mice

**Fig. 5:**
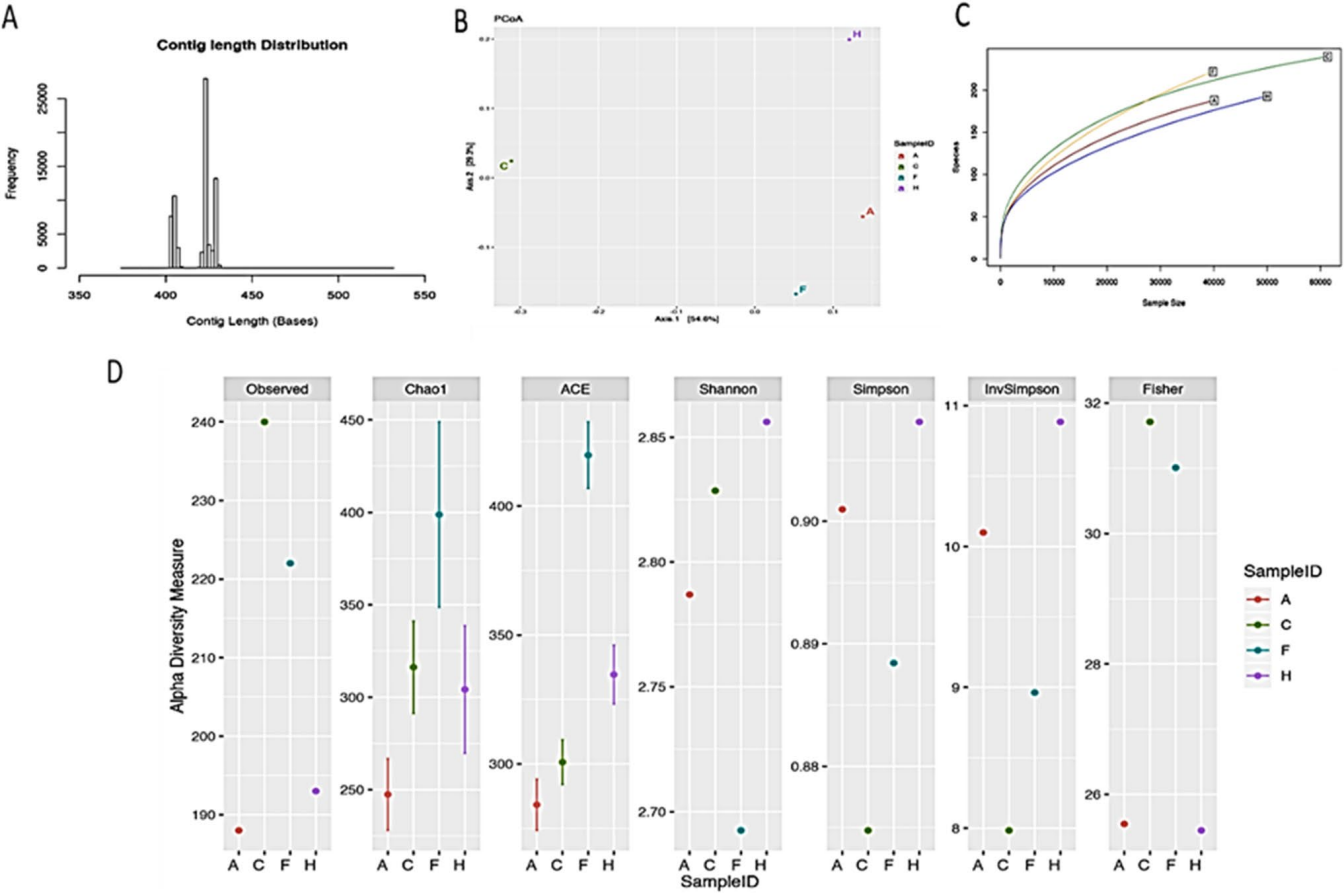
Effects of COS on the gut microbiota composition in mice with DSS-induced colitis. **A**) Histogram representing contig length distribution. **B**) Rarefaction curve. **C**) Principal component analysis (PCoA) plot. **D**) Alpha diversity measurements (Sample ID: A - DSS, C-Vehicle, F- DSS + Mesalamine and H- DSS + COS)

The impact of COS^Low^ on the gut microbiota in the DSS-induced IBD model was evaluated with 16S rRNA sequence analysis. The short reads of a high-quality assembled genome revealed 25,000 contigs with a length of 430 nucleotides were measured from the stool samples (Fig. 5A). The measure of variation recorded by a given number of reads in each group was recorded in Fig. 5B. As compared to the DSS treated group, the mesalamine treated and COS administered group demonstrated enrichment in microbial diversity (p<0.05). The principal component analysis (PCoA) of the taxonomical distribution revealed a lower spread in the DSS treated group as compared with the mesalamine and COS^Low^ treated groups (p<0.05) (Fig. 5C). The alpha diversity indices (A) Ace and (B) Chao1 reflect the OTU abundance in samples and Shannon (C) and Simpson (D) indices reflect the diversity of OTU in samples (Fig. 5D). The Simpson and Fisher values indicated richness and relative abundance in vehicle control, mesalamine control and COS^Low^ administrated groups as compared with the DSS treated group.

**Fig. 6:**
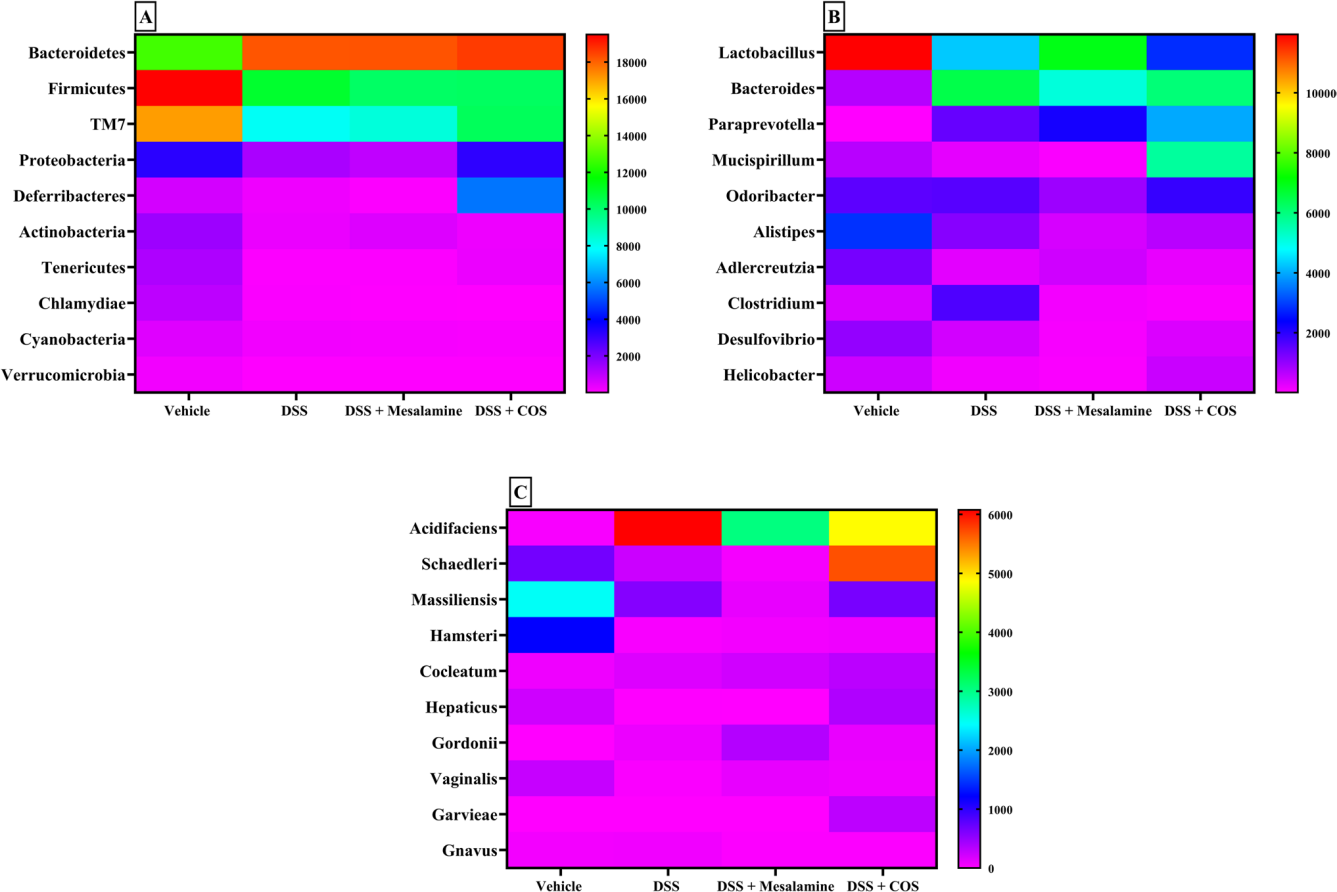
Effects of COS on the gut microbiota composition in mice with DSS-induced colitis. **A**) Top 10 phyla **B**) genera **C**) species abundance among the groups

The heat maps depict the abundance of the gut microbiota at the phylum, genera and species level in the DSS, mesalamine and COS^Low^ treated groups (Fig. 6A-C). Bacteroidetes, Firmicutes, TM7, Proteobacteria, Deferribacteres and Actinobacteria phyla were found to be the most abundant. The phyla Firmicutes, TM7, Proteobacteria displayed differences (p<0.05) among vehicle control, DSS, mesalamine and COS^Low^ administered groups*.* The abundance of phylum Bacteroidetes differed between the vehicle control and COS^Low^ administered groups (p<0.05), whereas Proteobacteria varied in its abundance between mesalamine, and COS^Low^ administered groups (p<0.05).

**Fig. 7:**
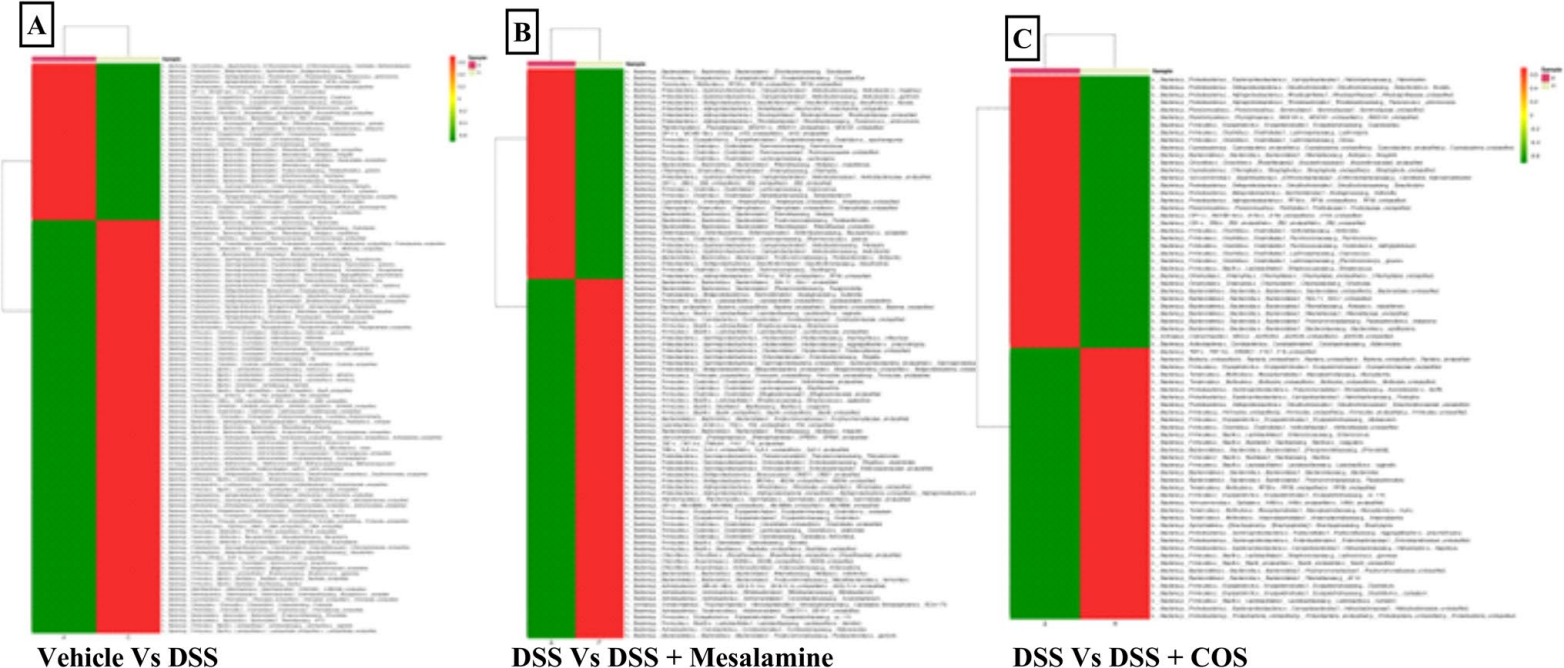
**A)** Heatmap representing the distribution of OTU in **A**) DSS vs. Vehicle comparison; **B**) DSS vs. Mesalamine comparison and **C**) DSS vs. COS comparison. Refer to supplementary Figure 2

*Paraprevotella, Mucispirillum* and *Odoribacter* were among the top 10 genera in terms of relative abundance, with variation in abundance between the mesalamine and the COS^Low^ administered group (p<0.05). The species *schaedleri*, *massiliensis*, *hamsteri* and *cocleatum* exhibited significant differences in numbers (p<0.05) between control, DSS, mesalamine and COS^Low^ administered groups. Fisher’s exact test was carried out using STAMP to identify statistically significant differences in OTU abundance between two samples (Fig. 7A-C). When the mesalamine group was compared with the vehicle control, there were 109 OTU that displayed variation in abundance (p<0.05). When the mesalamine treated group was compared with the DSS group, 92 significant OTU emerged (p<0.05). The mesalamine treated group when compared with the COS administered group resulted in 72 different OTU (p<0.05).

## Discussion

COS have always caught the attention of researchers as a class of naturally occurring, small compounds generated from marine sources with potential use in the functional food and pharmaceutical sectors (Joseph et al. 2021). Because of its anti-inflammatory effects, COS has received attention as a functional diet supplement (Hao et al. 2021). In the present study, we tested the hypothesis whether chronic COS^Low^ administration ameliorated DSS-induced chronic colitis in mice. Chronic colitis was induced by three repeated administration cycles of DSS (4%), with each cycle consisting of 5 days of DSS followed by 5 days of water. The COS administration was carried out consistently for a 30-day modelling period (Chassaing et al. 2014b).

Balb/c mice are frequently used for immunological studies that demonstrate TH2-mediated immune response, develop altered gut microbial composition and associated disease severity (Mukhopadhyay et al. 2022). Literature also supports the development of a translationally relevant model of chronic colitis using BALB/c mice (Hoffmann et al. 2018). Thirty days of COS^Low^ administration in BALB/c mice with chronic ulcerative colitis showed a significant protective effect with respect to changes in weight and DAI.

Although, the effect of COS^Low^ on histopathological scoring was found insignificant, the decreased inflammatory infiltration was observed in colon histopathology. Overall, the results suggested that COS administered even at low dose i.e. 20 mg/kg was able to show long-term efficacy against chronic colitis.

Increased levels of crucial proinflammatory cytokines, such as IL-6 and IL-1β, were evident in a translational model of DSS-induced colitis in BALB/c mice (Hoffmann et al. 2018). In the present study, colonic inflammation was evident in mice with DSS-induced chronic colitis and elevated levels of IL-6 and IL-1β (Mudter and Neurath 2007; Coccia et al. 2012). Treatment with COS^Low^ effectively reduced cytokine levels and suggested a protective effect in chronic colitis.

The epithelial restoration process is a vital phase in the healing of the intestinal lumen and plays a crucial role in maintaining intestinal barrier integrity in the face of inflammation. In the current work, the number of proliferative cells in colonic crypts of mice with chronic colitis was reduced in standard drug mesalamine, however COS^Low^ did not exert any significant effect on these changes. This indicates that at the dose tested (20mg/kg), COS in spite of protective against chronic colitis, fails to restore the epithelial changes in the intestine. These findings contrast with the earlier reports where COS at 20 mg/kg dose showed protection against DSS-induce chronic colitis (Yousef et al. 2012), may be due to the difference in the molecular weight of COS tested.

The above findings were corroborated further by the gut microbiota profiles in disease and treatment groups. In this study, the 16S rRNA gene sequence was used to analyse the gut microbiota in the vehicle control, DSS, mesalamine, and COS treated groups. The findings revealed that the intestinal microbiota in the vehicle control group was more diverse than that in the three test groups (Liu et al. 2020). The prevalence of species of bacteria in the intestinal microbiota was assessed using OTU of species of bacteria diversity and richness which are regarded as key markers of a "healthy" intestinal microbiome. Firmicutes, Bacteroidetes, Actinobacteria, Proteobacteria, Verrucomicrobia, Cyanobacteria, TM7, Fusobacteria, and Spirochaetes bacteria colonise the mouse distal gut, which is consistent with bacteria's role in maintaining the dynamic balance of the intestinal micro-ecosystem and ensuring normal physiological functions (Li et al. 2018). This is consistent with the results in our study, which show a more pronounced representation of the bacterial phyla Bacteroidetes, Firmicutes, Proteobacteria, TM7 and Deferribacteres.

In the study of the pathophysiology and therapeutics of IBD, gut microbiota has emerged as a crucial factor. Firmicutes and Bacteroidetes*,* the two most important bacterial phyla in the gastrointestinal system, have garnered considerable attention in recent years. It is believed that a healthy Firmicutes/Bacteroidetes (F/B) ratio is essential for preserving gut homeostasis. A change in the Firmicutes/Bacteroidetes (F/B) ratio, which indicates dysbiosis in the intestinal microbiota (Stojanov et al. 2020). An increasing number of studies have highlighted that a relative abundance decrease in Firmicutes and increase in Bacteroidetes indicate increased intestinal permeability and dysbiosis (Wu et al. 2018; Stan et al. 2020). A study conducted on mice treated with *Bacteroides ovatus* (*B. ovatus*) had a significant lack of cytokine production, including the anti-inflammatory cytokine. These results suggest that treatment with *B. ovatus* appears to inhibit or block the cytokine-driven response to DSS (Ihekweazu et al. 2019). In the current study, standard mesalamine and COS^Low^ treated groups displayed an improved F/B phyla ratio compared with DSS groups. The relative abundance of Bacteroidetes and Firmicutes increased and decreased, respectively, in DSS treated group*.* On comparing the COS administered group with the DSS group, we found that the F/B ratio increased, which suggests that intestinal permeability and dysbiosis decreased.

Proteobacteria appeared to be overrepresented in activities that contribute to the maintenance of the gut's inert atmosphere for the function of the microbiome by consuming oxygen and lowering the oxidation state in the intestinal environment. Bacteria in the Proteobacteria phylum are thought to play an important role in preparing the gut for the colonialization of strict anaerobes, which are required for proper gut function (Moon et al. 2018). These results are consistent with our study, in which the abundance of Proteobacteria was lower in the DSS group than in the vehicle group. This suggests that COS^Low^ may help to restore proper gut function through increasing the relative abundance of bacteria in the phylum Proteobacteria*.*

Many studies have emphasised Actinobacteria in the recent decade, particularly their function in gastrointestinal and systemic disorders. In spite of a small representation in the gut microbiota, they were found to be critical in maintaining gut homeostasis (Binda et al. 2018). In the present study, we observed an increase in the Actinobacteria diversity in the COS^Low^ group as compared with the DSS treated and mesalamine administered groups.

*Bacteroides fragilis*, a common human commensal, is reported to protect animals with colitis from developing intestinal inflammatory disorders. *B. fragilis* produces the immunomodulatory chemical polysaccharide A (PSA), which causes an anti-inflammatory immune response in intestinal tissue (Lee et al. 2018). The Bacteroides genus, which is part of the Bacteroidetes phylum, produces short chain fatty acids that increase the population of colonic Treg cells by promoting the movement of Treg cells from outside the intestines. Certain Bacteroidetes species manage inflammation via zwitterionic capsular polysaccharides, which are bacterial components that regulate T cells. These components have the ability to induce Treg cells, which secrete the anti-inflammatory interleukin-10, thereby contributing to immune regulation(Nomura et al. 2021).Consistent with the role reported by other groups, we observed an increase in *B. fragilis* abundance in the COS^Low^ administered group compared with the DSS and mesalamine treated groups.

The incidence of *Bacteroides acidifaciens* and *Christensenella minuta* is significantly lower in patients with rheumatoid arthritis, which suggests a potential microbial link for inflammatory arthritis and IBD (Xu et al. 2020). In the present study, we also found that *Bacteroides acidifaciens* increased in the COS^Low^ administered group compared with the DSS treated and mesalamine treated groups.

The presence of *Odoribacter splanchnicus* (*O. splanchnicus*) as a transmissible strain is linked to a reduction in inflammation in the intestines. Colonisation by *O. splanchnicus* causes an increase in regulatory T cells, which are identified by the expression of Foxp3+/ROR+. This colonisation also causes the production of short-chain fatty acids (SCFAs) (Saleh et al. 2023). In the present study, we also found that *Odoribacter* increased in the COS^Low^ administered group compared with the DSS treated and mesalamine treated groups.

In summary, the present study shows the protective effects (reduction of weight loss and disease severity evident from the morphological and histological examination of colons) of COS in a translational mouse model of chronic colitis. Furthermore, these findings were substantiated with evidence of a decline in colonic proinflammatory cytokines. In terms of the relationship between COS effectiveness and intestinal flora profile, the current study found that COS^Low^ inverted the abundant source of Firmicutes, TM7, Proteobacteria, and Deferribacteres gut microbiota while preserving microbiota diversity in the guts of mice with chronic UC. COS also significantly increased the population of TM7 and Deferribacteres. The administration of COS helped maintain the F/B bacterial ratio, which is instrumental in maintaining gut homeostasis. Hence, this study can be extended to understand the pathways of inflammation and the inflammasome, which are downregulated with the augmentation of the microbial population.

### Supplementary Information

Below is the link to the electronic supplementary material.

Supplementary file1 (DOCX 2609 KB)

## Data Availability

The authors confirm the availability of all data upon reasonable request.
