# Supplementary material for: Effect of chronic low-dose treatment with chitooligosaccharides on microbial dysbiosis and inflammation associated chronic ulcerative colitis in Balb/c mice
Source: Naunyn Schmiedebergs Arch Pharmacol. 2023 Sep 11;397(3):1611–22. doi: 10.1007/s00210-023-02710-3 (PMC10858833; doi:10.1007/s00210-023-02710-3)

Supplementary Figure 1. Representative images of colons

**
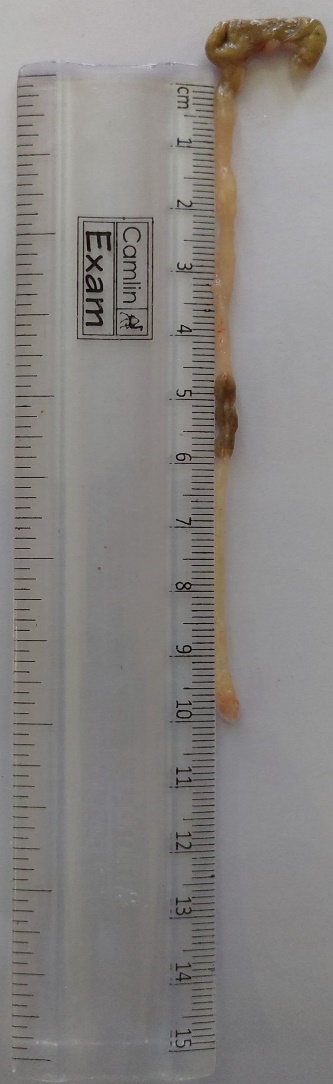

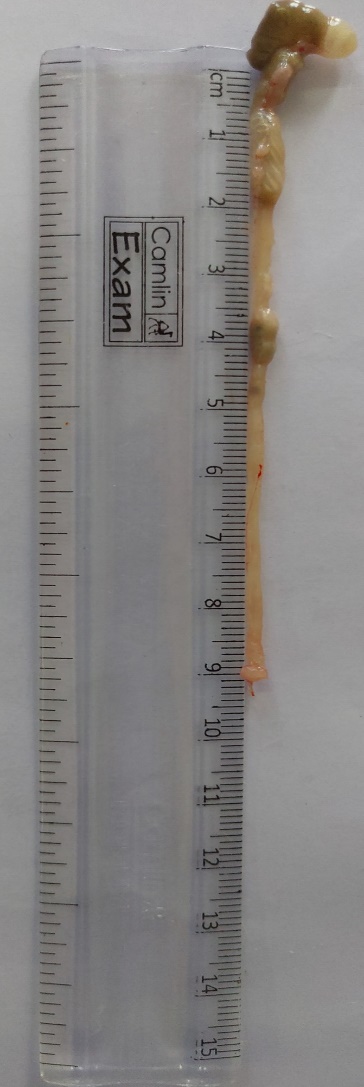

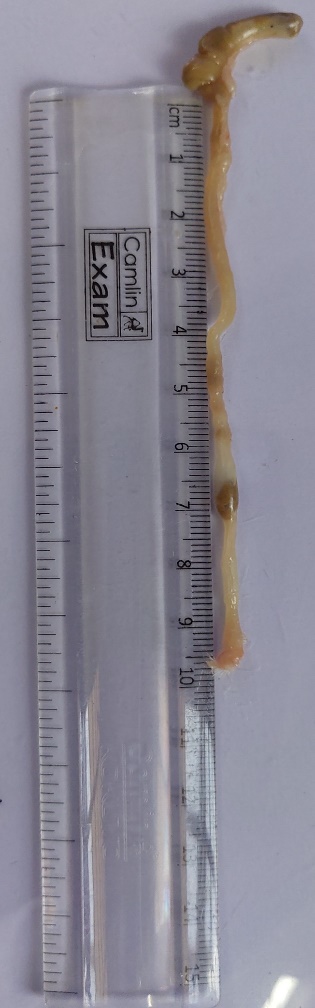

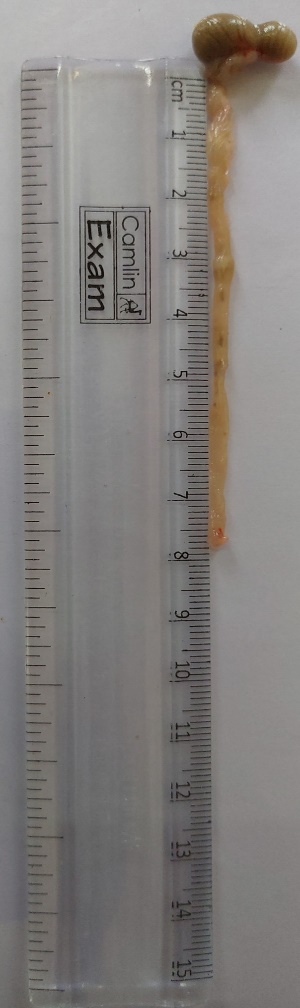

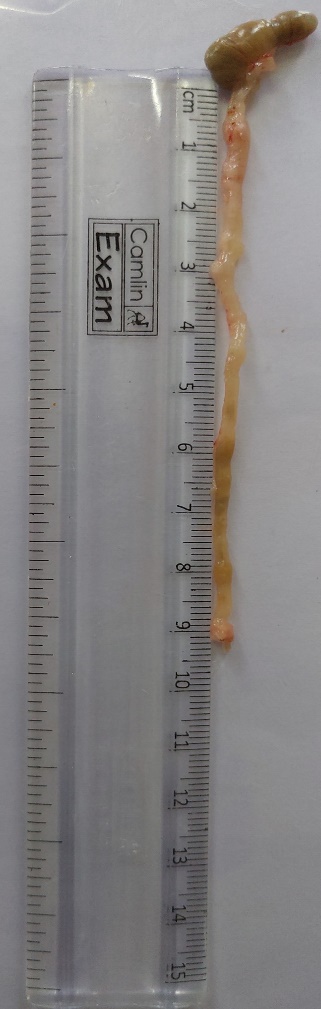

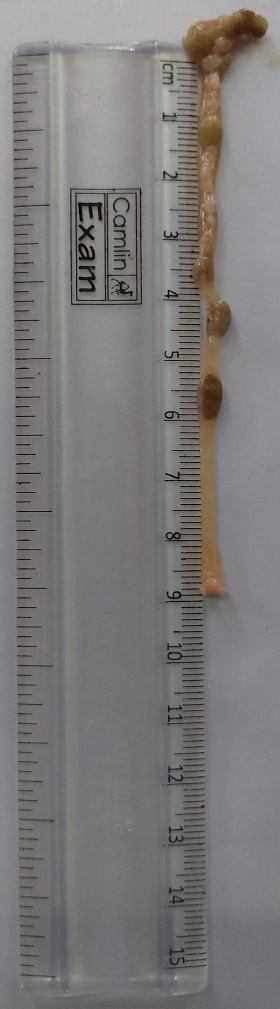
Vehicle:**

**DSS:**

**
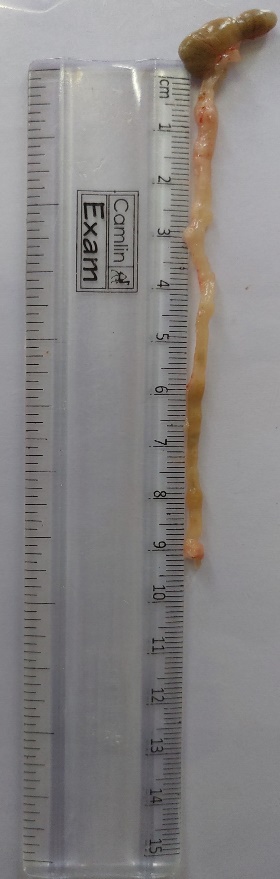

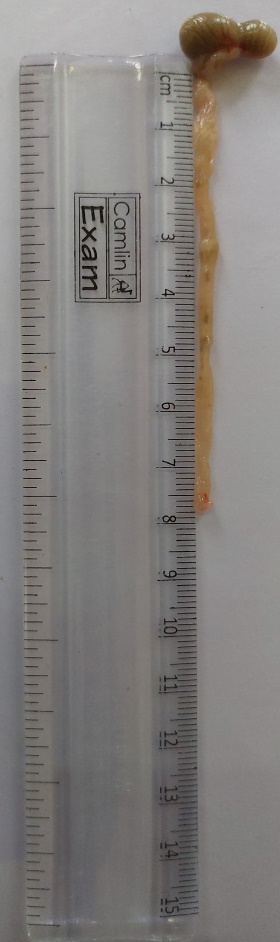

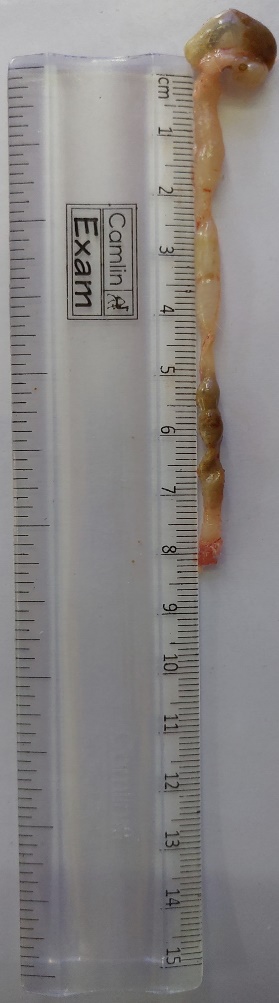

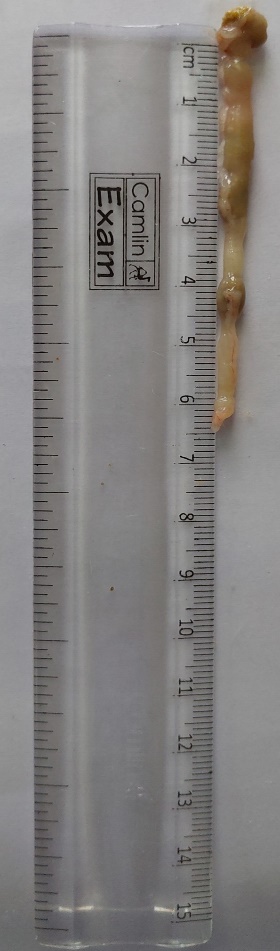
**

**DSS + Mesalamine** (Three colons out of 4 were photographed)

**
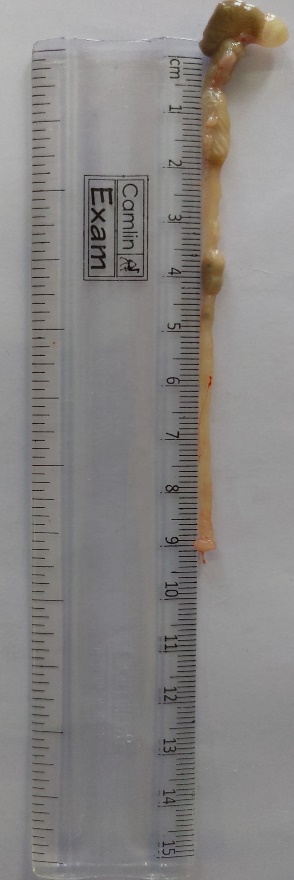

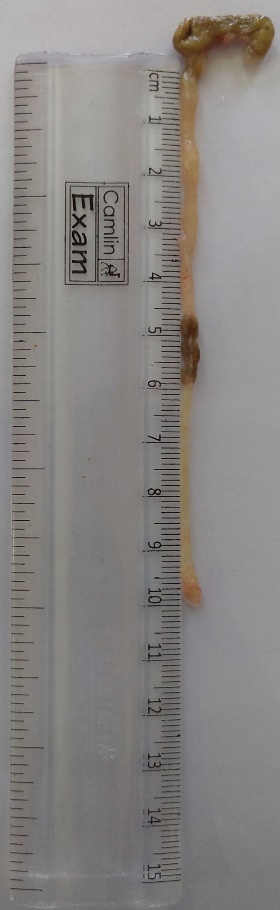

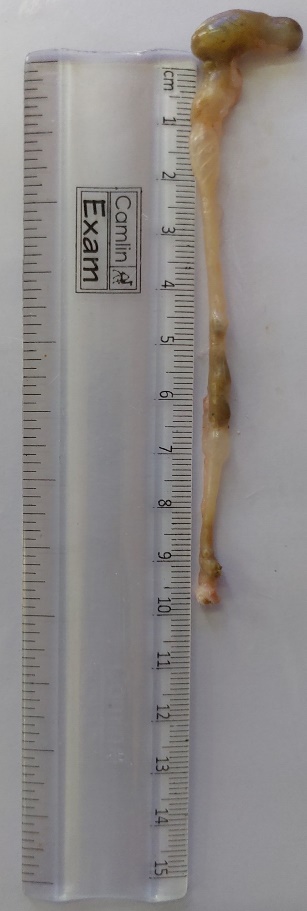
**

**
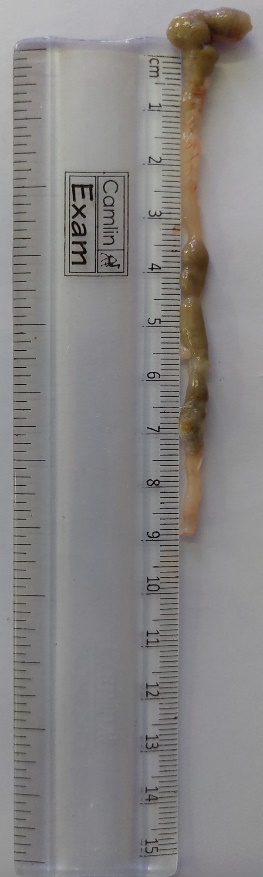

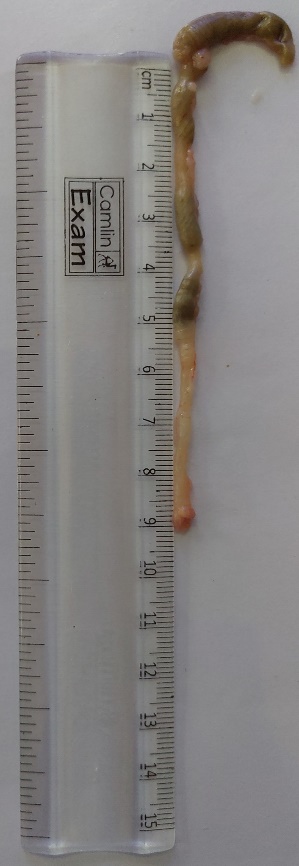
DSS + COS**

**
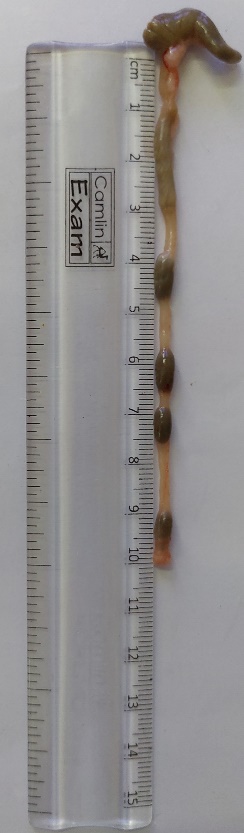
**
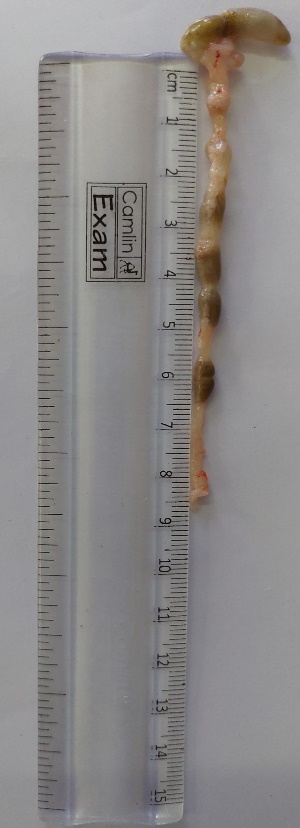


Supplementary Figure 2. Gut microbiota comparison

**Vehicle Vs DSS**


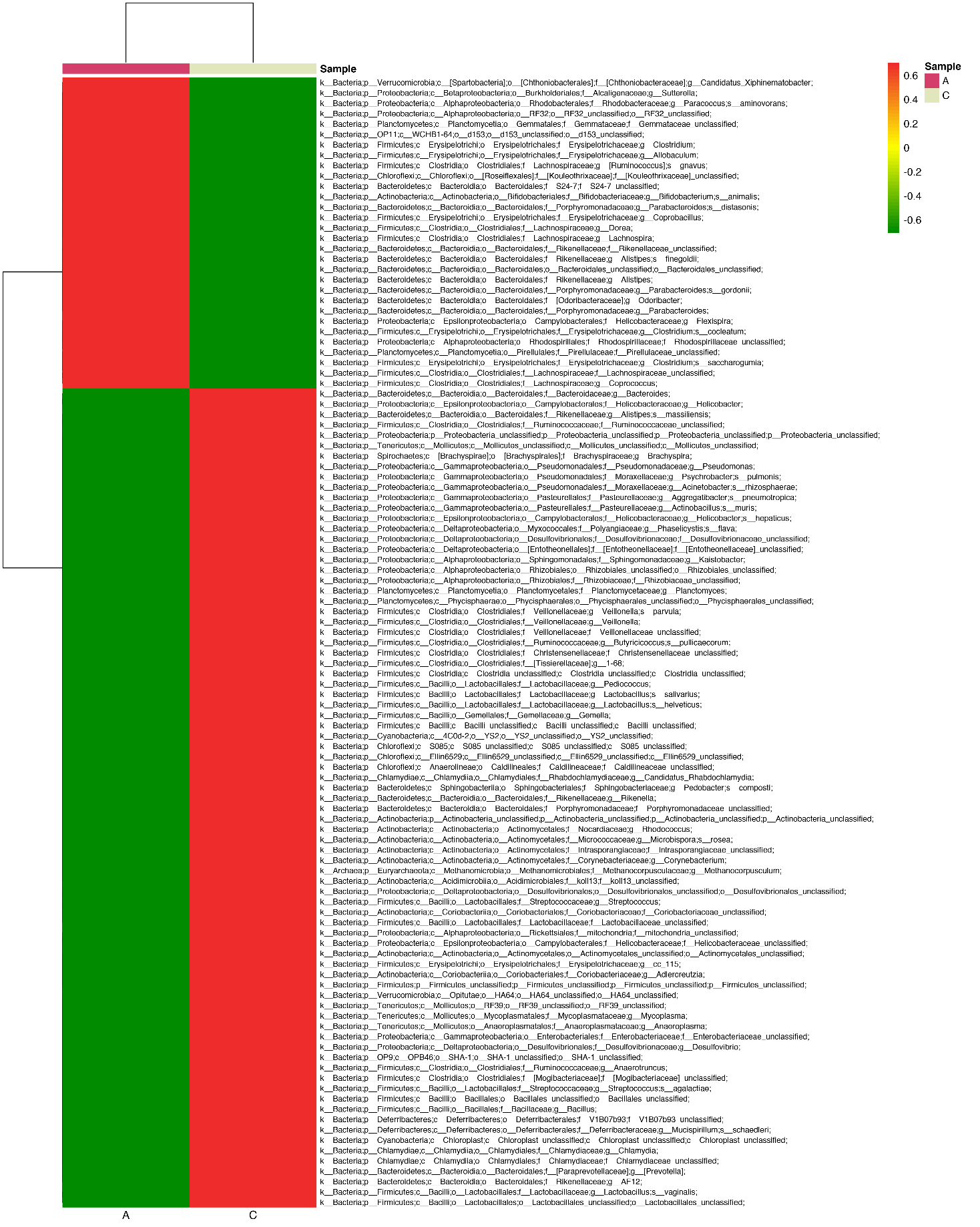


**DSS Vs DSS + Mesalamine**


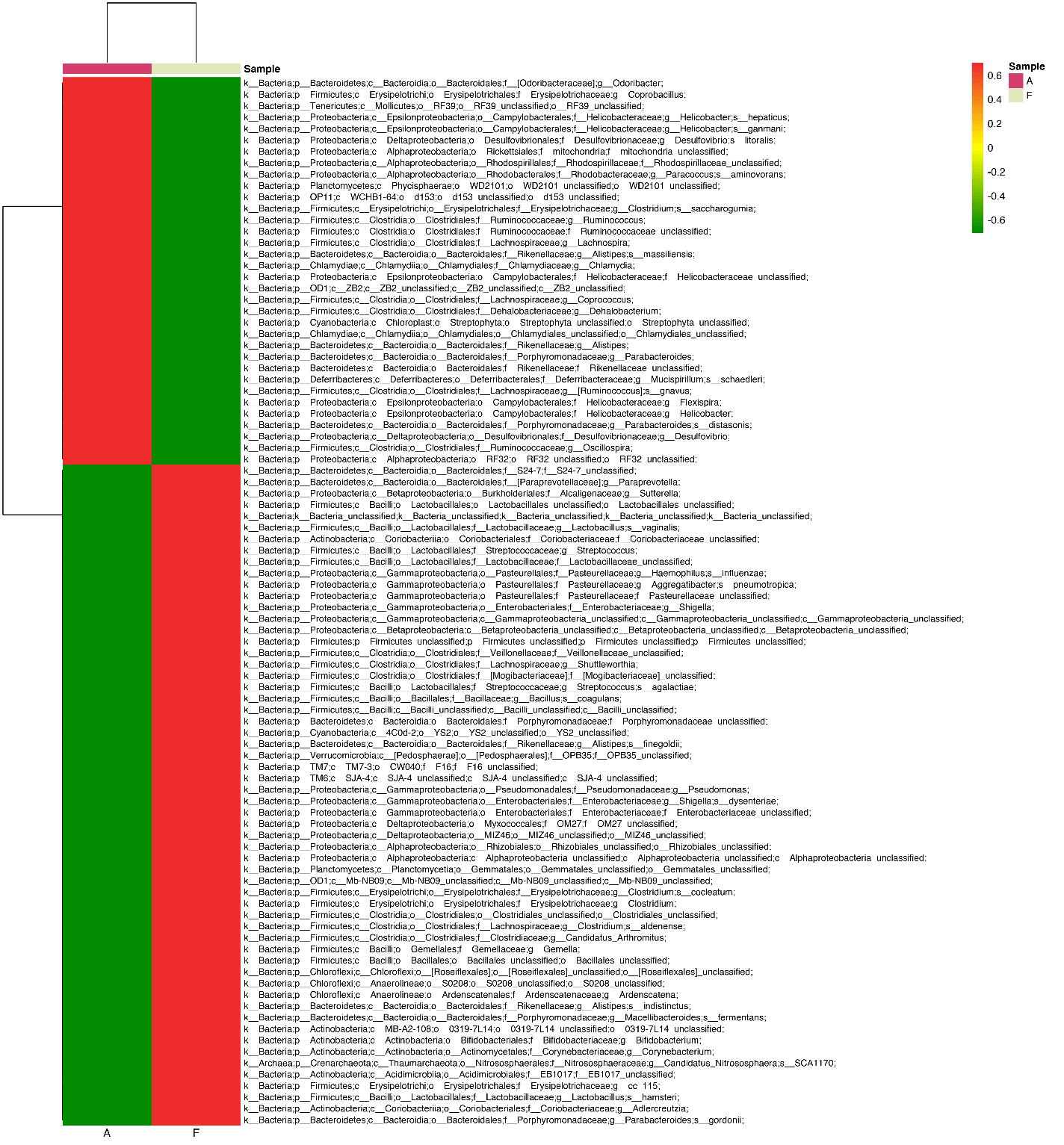


**DSS Vs DSS + COS**


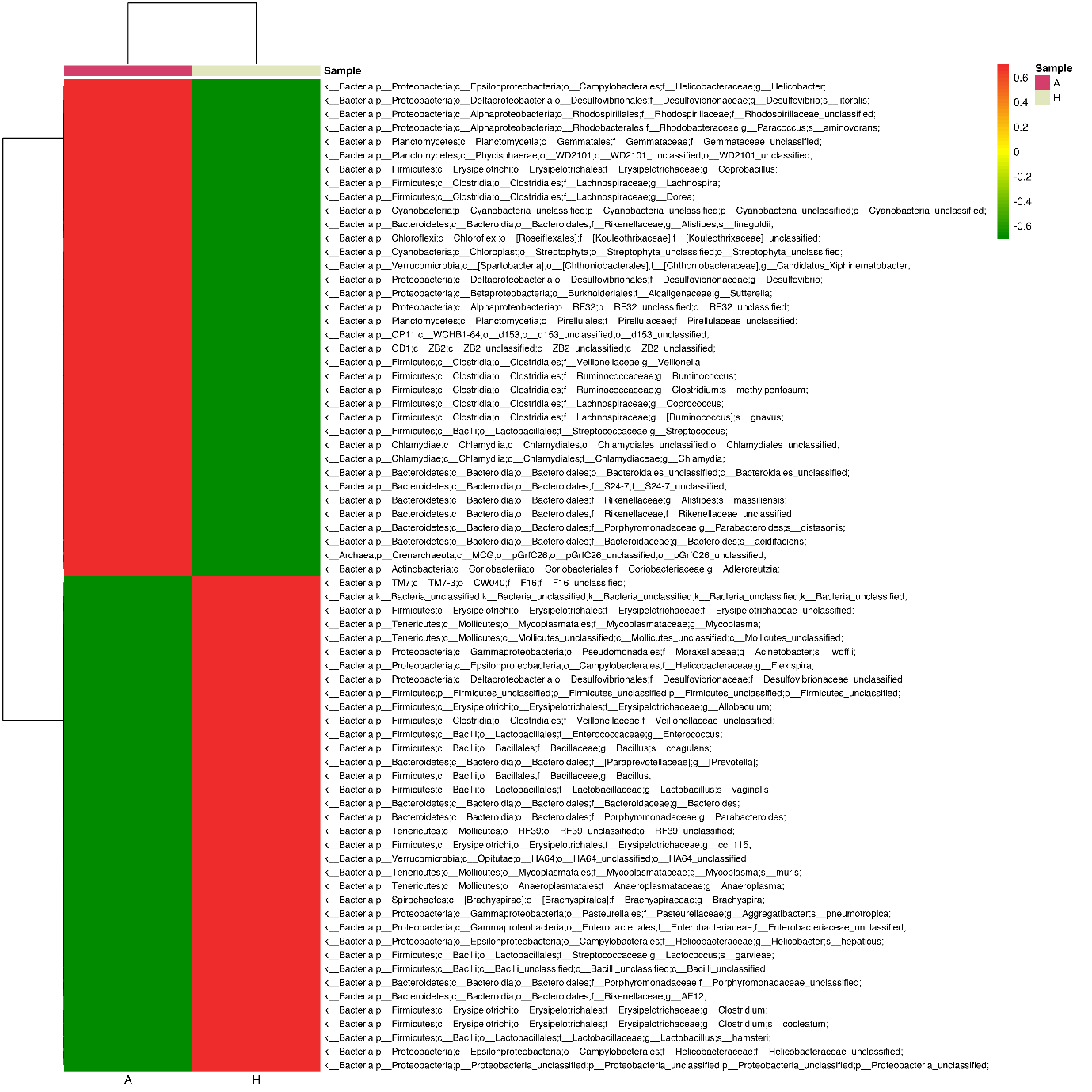

Supplement: Supplementary file 1 — Supplementary file1 (DOCX 2609 KB) [file 210_2023_2710_MOESM1_ESM.docx]
